# Disentangling Biological Transformations and Photodegradation Processes from Marine Dissolved Organic Matter Composition in the Global Ocean

**DOI:** 10.1021/acs.est.3c05929

**Published:** 2023-12-08

**Authors:** Sarah K. Bercovici, Maren Wiemers, Thorsten Dittmar, Jutta Niggemann

**Affiliations:** ‡ Institute for Chemistry and Biology of the Marine Environment (ICBM), School of Mathematics and Science, Carl von Ossietzky Universität Oldenburg, Ammerländer Heerstraße 114-118, Oldenburg 26129, Germany; § National Oceanography Centre, European Way, Southampton SO14 3ZH, Hampshire, United Kingdom; ⊥ Helmholtz Institute for Functional Marine Biodiversity (HIFMB), Carl von Ossietzky University of Oldenburg, Carl-von-Ossietzky-Straße 9-11, Oldenburg 26129, Lower Saxony, Germany

**Keywords:** dissolved organic matter, dissolved organic carbon, photodegradation, bioformation, biotransformation, molecular index, Fourier transform ion cyclotron resonance mass spectrometry, marine carbon cycle, organic carbon cycle

## Abstract

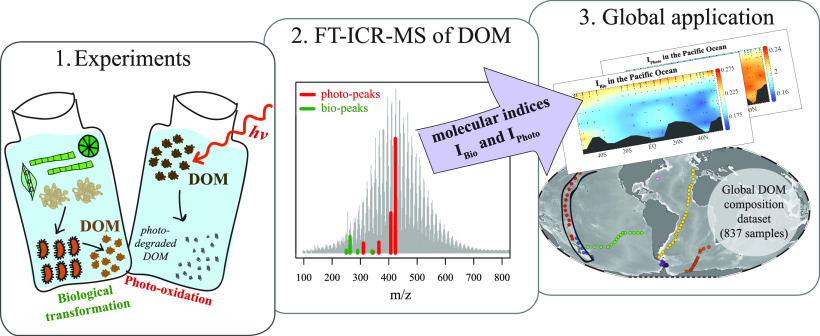

Dissolved organic matter (DOM) holds the largest amount of organic carbon in the ocean, with most of it residing in the deep for millennia. Specific mechanisms and environmental conditions responsible for its longevity are still unknown. Microbial transformations and photochemical degradation of DOM in the surface layers are two processes that shape its molecular composition. We used molecular data (via Fourier transform ion cyclotron resonance mass spectrometry) from two laboratory experiments that focused on (1) microbial processing of fresh DOM and (2) photodegradation of deep-sea DOM to derive independent process-related molecular indices for biological formation and transformation (*I*_bio_) and photodegradation (*I*_photo_). Both indices were applied to a global ocean data set of DOM composition. The distributions of *I*_photo_ and *I*_bio_ were consistent with increased photodegradation and biological reworking of DOM in sunlit surface waters, and traces of these surface processes were evident at depth. Increased *I*_bio_ values in the deep Southern Ocean and South Atlantic implied export of microbially reworked DOM. Photodegraded DOM (increased *I*_photo_) in the deep subtropical gyres of Atlantic and Pacific oceans suggested advective transport in warm-core eddies. The simultaneous application of *I*_photo_ and *I*_bio_ disentangled and assessed two processes that left unique molecular signatures in the global ocean.

## Abstract 2

Two novel indices, derived from molecular formulas detected via ultrahigh-resolution mass spectrometry, are used to assess photodegradation and biological transformation in dissolved organic matter across the global ocean.

## Introduction

Marine dissolved organic matter (DOM) represents one of the largest active reduced carbon reservoirs on Earth (662 ± 32 Gt C^1^). The most reactive pool of DOM is the labile DOM pool, which is primarily produced at the ocean surface via photosynthesis.^2^ While most of this DOM is incorporated into the cell biomass of heterotrophs or remineralized to CO_2_ via respiration,^1^ a fraction of it is transformed by both biotic and abiotic processes prior to advection into the deep. Refractory DOM comprises 95% of the ocean’s dissolved organic carbon (DOC) pool and remains in the deep ocean for centuries to millennia, mostly unavailable for immediate biological turnover.^3,4^ Reasons for this observed long-term stability are poorly understood and underlying processes are still under discussion.^5−9^

Processes adding or removing molecules from the marine DOM pool include photochemical^10^ and microbial transformations.^9,11−14^ Moreover, advection into deep water masses,^1^ aggregation into gels and colloidal material,^15^ adsorption and desorption to and from particles,^16^ and solubilization of sinking particles^17^ are processes that can introduce surface-derived DOM to depth. These processes all render DOM into a vast, molecularly diverse pool of organic carbon in the deep ocean.^9^

Even though DOM forms the basis of microbial life in the ocean, more than 90% of it resides in the deep sea, resisting biological utilization for centuries to millennia.^1^ For understanding the conversion of DOM from highly reactive (labile) to mostly unreactive (refractory), it is important to identify the involved processes and assess their relative impact on the molecular composition of DOM. These processes include biological reworking (formation, transformation, degradation, remineralization) and chemical alterations (i.e., oxidation, polymerization, condensation). These processes all are influenced by prevailing environmental conditions (e.g., nutrient levels, salinity, temperature, and light) and act simultaneously on the DOM pool on different temporal and spatial scales. Therefore, studies of specific processes are mostly restricted to controlled laboratory experiments with limited time spans.^11,14^ Furthermore, the complexity of marine DOM makes its detailed chemical characterization challenging. The advent of ultrahigh resolution analytical techniques such as Fourier-transform ion cyclotron resonance mass spectrometry (FT-ICR-MS) enables attainment of molecular information on the DOM composition in unprecedented detail. While fully understanding the metabolic pathways responsible for the conversion of labile to refractory DOM would require elucidating the molecular and isometric structures of intermediate products, with FT-ICR-MS, it is possible to resolve and detect thousands of molecular formulas. It should be noted, however, that each molecular formula identified in FT-ICR-MS corresponds to many isomers, so one DOM sample contains tens of thousands of molecular formulas, corresponding to hundreds of thousands of compounds.^18^ As such, FT-ICR-MS provides a fingerprinting method to assess molecular patterns associated with specific processes but does not go into detail on the isomeric composition of DOM; that would require further purification steps and analytical techniques, such as nuclear magnetic resonance spectroscopy.

Since photodegradation is an abiotic process, identifying molecular formulas that are added and removed with sunlight exposure in the marine environment is relatively straightforward. Assessing biological formation and transformation, however, is more complicated. Previous studies have shown that single bacterial strains release very different DOM depending on growth conditions.^19−21^ However, laboratory experiments with more complex microbial communities demonstrate microbial transformation of bioavailable substrates into DOM that is notably like natural marine DOM.^12,22^ Moreover, there are universal structures within DOM that are observed in diverse environments (i.e., fresh vs marine water, surface vs depth).^23^ For instance, carboxylic-rich alicyclic moieties (CRAM)^24^ and material derived from linear terpenoids^25^ are a common structural feature and have been chemically characterized as molecularly recalcitrant DOM constituents. Additionally, characterizing labile DOM with a boundary on H/C ratios is applicable to a diverse data set collected over the span of a decade.^26^ Even in diverse systems, there are universal molecular signatures that provide insights toward lability or recalcitrance of DOM from a given environment. Our approach in this study assumes that microbially produced DOM from a laboratory experiment shares characteristics with DOM in the natural environment, independent of community composition, available substrates, and prevailing growth conditions. As such, we hypothesize that the processes shaping the DOM composition in incubation experiments are representative for the natural open ocean environment and that the results from the laboratory study can be scaled up to global dimensions.

Past studies have derived other indices for marine DOM to describe its state and its sources. The degradation state of DOM can be assessed by the amino acid based degradation index that links systematic changes in amino acid composition to the reactivity of bulk organic matter.^27^ Flerus et al.^28^ introduced a degradation index (*I*_deg_) based on the molecular fingerprints of DOM obtained via FT-ICR-MS. They correlated intensities of mass peaks in marine DOM samples with the radiocarbon age of the respective sample and identified peaks systematically increasing and decreasing in intensity with age. Previous studies applied the degradation index on largescale environmental data from the Atlantic,^28,29^ Southern^30^ and Pacific^31,32^ oceans. Medeiros et al.^33^ derived the terrigenous DOM index through identifying 184 molecular formulas that are indicators of riverine inputs into the ocean.

While all these indices consider characteristics of bulk DOM (degradation state, lability, and terrestrial contribution), they do not provide information on the processes that shape DOM composition. Gomez-Saez et al.^34^ developed an index to assess the extent of abiotic sulfurization, with 15 molecular formulas identified as exclusively produced by abiotic sulfurization of DOM. Apart from this recently introduced index, there are no published indices that provide process-specific information. In this study, we develop two indices to distinguish biological transformations and photochemical degradation, two major processes that affect the global DOM pool in the natural environment.

Photochemical and biological transformations both have their maximum impact in the sun-lit, warm, and productive surface layer. Moreover, these processes can be mutually dependent.^35−37^ Photo-oxidation of surface ocean DOM can either lead to an enhanced or decreased biological availability for some DOM.^38,39^ Photo-oxidation of upwelled deep waters is a proposed mechanism for the radiocarbon depletion of nucleic acids in open ocean bacteria; photochemistry converts the old, recalcitrant DOM into a more bioavailable form.^40^ Moreover, photochemical production of aromatic compounds can enhance the microbial consumption of DOM.^41^ Biological production might also influence photodegradation. For instance, higher particle density production could cause a shading effect and lower the photodegradation potential. Furthermore, algae produce a variety of photoprotective compounds that act as antioxidants or absorb UV radiation,^42^ affecting both the bioavailability of organic matter and its susceptibility to photochemical degradation.

In this study, we introduce two process-specific indices to distinguish and assess bioformation and transformation (*I*_bio_) and photodegradation (*I*_photo_) in natural DOM samples, derived from molecular data obtained via FT-ICR-MS. The two indices disclose the respective process driving the observed molecular changes. We applied the newly developed indices to extensive DOM data sets comprising 837 samples from the Atlantic, Southern, and Pacific oceans, demonstrating that both process-related indices are applicable to the marine environment on a global scale.

## Materials and Methods

### DOM Samples

The process-related indices introduced in this study were derived using data from a three year laboratory mesocosm experiment studying the natural microbial formation and reworking of DOM^22^ and a photodegradation experiment on North Atlantic Deep Water (NADW).^14^ For the mesocosm laboratory experiment, the authors mixed ∼10 L of artificial seawater with 100 mL of prefiltered (200 μm) coastal North Sea water inoculum. These mesocosms were incubated at room temperature on a 12:12 h light:dark schedule (the light was in the visible range, between 400–700 nm). After 167 days, 1.5 L of each mesocosm was filtered through 1.2-μm combusted glass fiber filters to remove aggregates and phytoplankton and then incubated in the dark for the remaining time of the 1011 day experiment. Subsamples for the experiment were collected at regular intervals. The bioformation and transformation index that we define in this work is from the molecular composition of DOM from the second year of the experiment.

The photochemical experiment was conducted from North Atlantic Deep Water collected from a CTD rosette via gravity flow at 3000 m from the Bermuda Atlantic Time Series (31°40′ N:64°10′ W) aboard the *RV Atlantic Explorer* in November, 2009. The water was transported back to the lab, where it was frozen until the photochemical experiment, when the samples were thawed and transferred to precombusted spherical quartz irradiation flasks. All samples were then placed under a solar simulator that mimicked natural sunlight from 295 to 365 nm. Samples were left in the simulator at a constant temperature for 28 days. This solar simulator is designed so that 1 day is approximately 1.27 times the daily solar irradiance during the winter at 36.89°N or 0.67 times the daily (12 h) irradiance at the equator.^14,43,44^

**Figure 1:**
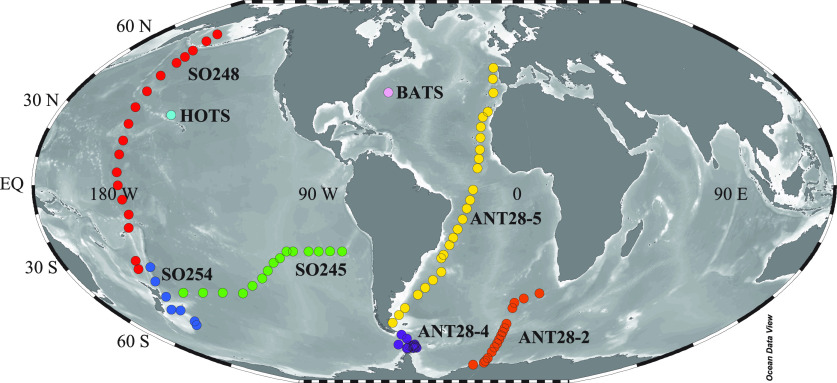
Cruise tracks in the Atlantic, Southern, and Pacific oceans. The sections in Figures 3 and 5 correspond to ANT28-5 (yellow) for the Atlantic transect, ANT28-2 (orange) for the Southern Ocean, and SO248 (red) and SO254 (blue) for the Pacific transect.

The indices developed from these two experiments were applied to 837 samples collected from the Atlantic, Southern, and Pacific oceans. Atlantic and Southern Ocean DOM samples were taken during three *R.V. Polarstern* cruises ANT-XXVIII/2 (Atlantic sector of the Southern Ocean; 39.2° S to 70.5° S), ANT-XXVIII/4 (Drake Passage and Antarctic Peninsula; 56.1°S to 62.4° S) and ANT-XXVIII/5 (Atlantic; 51° S to 47° N) in austral spring and summer (Dec 2011 - May 2012; Figure 1). Samples in the Pacific Ocean and Pacific sector of the Southern Ocean were likewise collected during three cruises aboard the *R.V. Sonne*: SO245 (subtropical South Pacific; 165° W to 95° W, between 40° and 20° S; December 2015 to January 2016), SO248 (Pacific latitudinal transect from 30° S to 50° N; May, 2016) and SO254 (Pacific Sector of the Southern Ocean between 50 and 30° S; January and February 2017) (Figure 1). All samples were collected from a rosette sampler via a gravity flow.

All samples (experimental and environmental) were extracted according to the solid-phase extraction (SPE-) method introduced by Dittmar et al.^45^ For the mesocosm experiment, 150–250 mL of from each time point were solid-phase extracted from filtered and acidified samples with 100 mg PPL columns (Agilent, USA).^22^ For the photochemistry experiment, SPE was conducted with ∼1.5 to 2 L of the acidified water samples extracted with 1 g PPL columns.^46^ For the environmental samples, four L of seawater were filtered through precombusted (400 °C, 4 h) 0.7 μm glass fiber filters (GF/F, Whatman, United Kingdom) and acidified to a final pH of 2 (HCl, 25%, p.a., Carl Roth, Germany). The samples were extracted on commercially prepacked cartridges (1 g of sorbent, PPL, Agilent, USA) via gravity flow. After extraction, the cartridges were deionized by rinsing with two cartridge volumes of ultrapure water (pH 2). The cartridges were then dried with nitrogen gas and immediately eluted with 6 mL of methanol (HPLC-grade, Sigma-Aldrich, USA) into precombusted amber vials. These DOM extracts were stored in the dark at −20 °C until further analysis in the laboratory. The carbon-based extraction efficiency was 53 ± 9% for Atlantic DOM, 69% for the mesocosm experiment,^22^ and 67–74% for the photodegradation experiment.^14^

### Molecular Composition of DOM

All DOM extracts were analyzed on a SolariX XR FT-ICR-MS instrument (Bruker Daltonik GmbH, Bremen, Germany) equipped with a 15 T superconducting magnet (Bruker Biospin, Wissembourg, France) and an electrospray ionization source (ESI; Apollo II ion source, Bruker Daltonik GmbH, Bremen, Germany). For analysis validation, an in-house reference sample (North Equatorial Pacific Intermediate Water (NEqPIW); www.icbm.de\en\ds-dom), collected at the Natural Energy Laboratory of Hawaii Authority in 2009,^47^ was measured regularly. FT-ICR-MS measurements were conducted in ESI negative ion mode, following the same method outlined in Bercovici, Dittmar and Niggemann, 2021.^48^ A summary of the compiled environmental data used here is available at 10.1594/PANGAEA.962747.^49^

### Bioformation and Transformation (*I*_bio_) and Photodegradation (*I*_photo_) Indices

The bio- formation and transformation index (*I*_bio_) was developed based on results of a three year mesocosm experiment on DOM production by a natural microbial community of phyto- and bacterioplankton.^22^ We used an integrated sample over the course of the second year of the mesocosm experiment, in which the DOM contained a mixture of freshly produced and microbially transformed DOM that meets the reactivity criteria for labile, semilabile, and semirefractory marine DOM with lifetimes of hours to decades.^2^

The photodegradation index (*I*_photo_) was derived from data obtained during a photodegradation experiment of North Atlantic Deep Water (NADW) sampled at the Bermuda Atlantic Time Series site (BATS).^14^ This photodegradation experiment used a solar simulator emitting high energy irradiance, with UV-light in the range of 295 to 365 nm, thus inhibiting microbial growth.^50^ Therefore, the dominant process shaping DOM composition in this experiment was photodegradation of natural DOM.

We used the NEqPIW reference sample^47^ for comparison to identify any bioformation and transformation or photodegradation-related changes in DOM composition. This reference material was not treated photochemically. Moreover, as deep sea DOM is considered refractory and stable for long time scales,^3,4^ this reference material represents DOM that is not freshly microbially produced. Therefore, mass peaks in the mesocosm (fresh) sample that showed a higher relative intensity than the deep sea sample were considered potential “marker peaks” for bioformation and transformation. All mass peaks in the spectra considered in this analysis exhibited a Gaussian-like distribution typical of the marine DOM.

Mass peaks selected for *I*_bio_ had to fulfill the following three criteria: First, selected peaks must have an intensity >5% of the peak with the highest intensity in the respective sample, which makes their occurrence more likely in a larger variety of environmental samples and reduces the variability in the calculated index. Second, the intensity of selected peaks in the integrated mesocosm sample must be at least 30% higher than that of the same peaks in the NEqPIW sample (normalized peak intensity; Table 1). Third, the selected peaks must be unsusceptible to photodegradation, hence their relative peak intensity in the molecular composition samples of the photodegradation experiment had to remain unchanged (Table 1, peaks B1–B5 in NADW before and after photodegradation were not significantly different, Welch two sample *t* test, *p* > 0.99).

**Table 1:** Selected Mass Peaks, Assigned Molecular Formulas, *m*/*z*, Peak Intensities for Untreated NADW, Photodegraded NADW, NEqPIW, and the Mesocosm for Each Selected Peak, and Factor of Peak Intensity Changes (*F*) between Untreated and Photodegraded NADW (P), between the Mesocosm and NEqPIW DOM (B) and between NEqPIW and both Photodegradation Experiment and Mesocosm^a^

				Photodegradation experiment		Mesocosm experiment		
	Peak	Molecular formula	*m*/*z*	Peak intensity NADW	Peak intensity photodegraded NADW	Peak intensity NEqPIW	Peak intensity mesocosm	*F*
Photodegradation	P1	C_16_H_16_O_7_	319.0823	0.3164	0.1885	0.3596	0.3880	0.60
	P2	C_19_H_20_O_7_	359.1136	0.2970	0.1990	0.4039	0.4047	0.67
	P3	C_16_H_20_N_2_O_9_	383.1096	0.2553	0.1774	0.2693	0.2571	0.69
	P4	C_23_H_26_O_8_	429.1555	0.2675	0.1797	0.2767	0.2664	0.67
	P5	C_24_H_30_O_8_	445.1868	0.4455	0.2985	0.4110	0.4290	0.67
bioformation and transformation	B1	C_13_H_18_O_5_	253.1081	0.3199	0.3094	0.3697	3.8675	10.46
	B2	C_13_H_16_O_6_	267.0874	0.3695	0.3713	0.4272	1.8540	4.34
	B3	C_13_H_19_NO_6_	284.1140	0.0789	0.0800	0.1030	0.6412	6.22
	B4	C_13_H_17_NO_7_	298.0932	0.1403	0.1408	0.1890	0.5933	3.14
	B5	C_18_H_28_O_7_	355.1762	0.4434	0.4528	0.5285	2.1822	4.13
	D1	C_14_H_16_O_8_	311.0772	0.5165	0.5197	0.6140	0.6512	1.03
	D2	C_17_H_24_O_8_	355.1398	2.1211	2.1366	3.0489	3.0650	1.00
	D3	C_16_H_22_O_9_	357.1191	1.4340	1.4880	1.9826	1.8309	0.94
	D4	C_19_H_28_O_9_	399.1661	1.4453	1.3841	1.7431	1.8609	1.06
	D5	C_19_H_28_O_10_	415.1610	1.0417	1.0112	1.2703	1.3158	0.99

a The factor of relative peak intensity change (*F*) was calculated by dividing the relative peak intensity of the photodegraded NADW by that of the untreated NADW for photodegradation and the relative peak intensity of the mesocosm by that of the NEqPIW for bioformation and transformation. The D peaks were neither influenced by photodegradation nor bioformation and their relative intensity remained constant in all samples. Calculations for *I*_bio_ and *I*_photo_ are given in eqs 1 and 2, respectively.

A similar set of conditions was met for mass peaks used to derive *I*_photo_. First, the selected mass peaks must have an intensity >5% of the maximum peak intensity in the respective sample. Second, the intensities of the selected mass peaks must be at least 30% lower in the irradiated sample than in the sample prior to irradiation. Third, the influence of bioformation and transformation on the selected mass peaks must be negligible, meaning that the normalized peak intensities in the mesocosm sample were not different in the NEqPIW reference sample (Table 1; peaks P1–P5 in the NEqPIW and mesocosm sample were not significantly different, Welch two sample *t* test, *p* = 0.92). While the presence of these photosusceptible peaks (P-peaks; Table 1) in the mesocosm sample implies that they are biologically formed, their persistence at a constant intensity in the reference sample suggests that they are not susceptible to immediate biological degradation.

## Results

### Index Development and Validation: Defining *I*_photo_ and *I*_bio_

**Figure 2:**
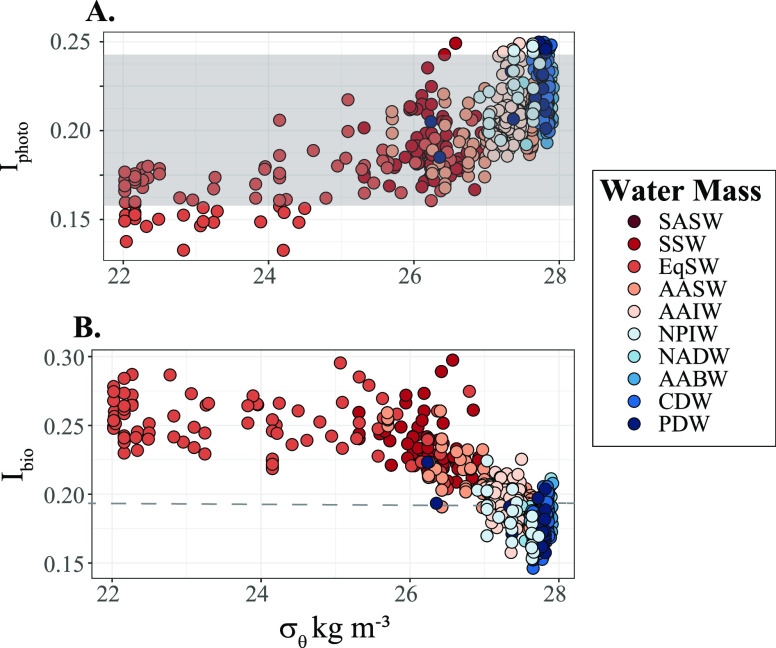
(A) *I*_photo_ and (B) *I*_bio_ for all samples (locations illustrated in Figure 1) along seawater density. The upper and lower boundaries of the gray box represent the *I*_photo_ of untreated and photodegraded NADW DOM (*I*_photo_ = 0.24 and 0.16, respectively). The lower dashed line represents the *I*_bio_ of NEqPIW DOM (*I*_bio_ = 0.19). Note that the *I*_bio_ of mesocosm DOM is 1.05 and therefore not shown on the *y*-axis. Water mass definitions are based on work from Schmitz^59^ and Talley^60,61^ for the open ocean basins and Orsi et al.^62^ for the Southern Ocean and Antarctic water masses. In brief, SASW is Subarctic Surface Water, defined as surface water (σ_θ_ < 27 kg m^–3^) in the subarctic regions (50 > latitude > 40 N and S, respectively). SSW is Subtropical Surface Water (between latitudes of 20 and 40 N and S). EqSW is equatorial surface water (between 20 S and 20 N). AASW is Antarctic Surface Water, beyond the southern polar front (50 S). AAIW is Antarctic Intermediate Water ((defined as σ_θ_ = 27 and salinities < 34; here also includes mode waters), which are colder, fresher water masses derived from AASW that fill the intermediate layer depths in both the lower latitude Atlantic and Pacific ocean basins. NADW is North Atlantic Deep Water, defined as the deep waters with θ > 2 °C and σ_θ_ = 27.7 kg m^–3^. AABW is Antarctic Bottom Water (defined as σ_θ_ > 27.7 kg m^–3^ and θ < 2 °C), which originates in the Southern Ocean and Antarctica and fills the ocean basins in the Atlantic Ocean and Atlantic Sector of the Southern Ocean. CDW is Circumpolar Deep Water, which is defined as the same criteria as AABW, but in the Pacific Ocean and Pacific Sector of the Southern Ocean. PDW is Pacific Deep Water, an old southward flowing water mass derived from overturning CDW. PDW is known for its high apparent oxygen utilization (AOU) and is defined as AOU > 100 μM and θ ≥ 2 °C. NPIW, or North Pacific Intermediate Water, is a relatively fresh intermediate water in the far north Pacific occurring between 300 and 800 m, likewise known for its high AOU (>250 μM).

For each index, we selected five mass peaks that met the criteria described above for each process (Table 1, P1 – P5 for *I*_photo_ and B1 – B5 for *I*_bio_). The rationale for selecting five mass peaks for each index calculation is to cover a maximum possible mass range and to be applicable in a maximum variety of different environments. The peaks selected for *I*_photo_ cover a slightly wider mass range (∼300–450 Da) than those selected for *I*_bio_ (∼250–360 Da). For the index calculation, the intensities of the five molecular formulas were summed up and divided by the sum of five molecular formulas that were neither influenced by photodegradation nor bioformation and transformation (peaks D1-D5) and had similar relative intensities in the integrated mesocosm sample (within 2%; Table 1), the photodegradation experiment samples, and the NEqPIW reference sample. The equations for the two indices are as follows:
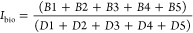
1
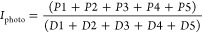
2A prerequisite for a universally applicable index based on mass spectrometric molecular data is the occurrence of the selected mass peaks in a wide variety of environments. As such, both *I*_bio_ and *I*_photo_ indices were applied to the global ocean data set (Figure 1). The *I*_photo_ values of untreated and photodegraded NADW DOM from the experiment^14^ were 0.24 and 0.16, respectively, illustrating that more photodegraded DOM holds lower *I*_photo_ values (Figure 2A). The *I*_bio_ was 0.19 for the NEqPIW sample and 1.05 for the integrated mesocosm sample (Figure 2B). The calculated indices for the endmembers of both processes are indicated as the upper and lower boundaries of the gray box and dashed line in Figure 2A and B, respectively.

### Index Application to Environmental Samples

**Figure 3:**
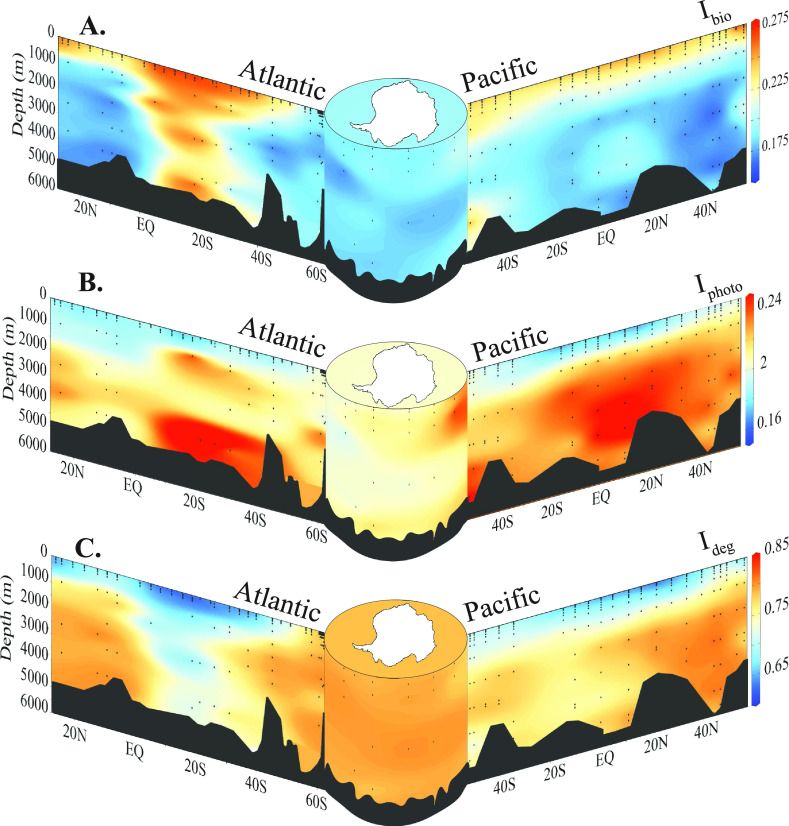
U-shaped plot of (A) the bioformation and transformation index (*I*_bio_), (B) the photodegradation index (*I*_photo_), and (C) the degradation index (*I*_deg_^28^) in the Atlantic, Southern, and Pacific oceans. Sections are defined using cruises SO248, SO254, ANT 28-5, and ANT28-2 (Figure 1).

**Figure 4:**
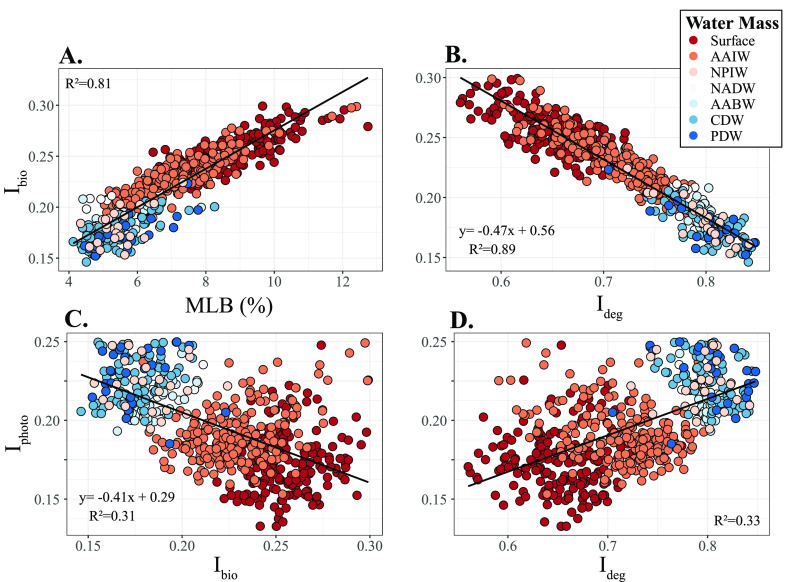
Correlation between *I*_bio_ and (A) molecular labile boundary (MLB; D’Andrilli et al., 2015), the index used to identify labile molecular formulas, and (B) *I*_deg_ (Flerus et al., 2012). Correlation between (C) *I*_photo_ and *I*_bio_ and (D *I*_photo_ and *I*_deg_. Colors correspond to water masses, which are described in the Figure 2 caption. The equations in subpanels B and C are used for the calculated anomalies plotted in Figure 5.

In general, *I*_photo_ values in the global ocean increased with increasing water depth and density (Figure 2A). Most (88%) of the *I*_photo_ values in the marine environment were within the boundary set by the photodegradation experiment (Figures 2A, 3A). The *I*_photo_ values in the global ocean were lowest in the surface mixed layer (0.13) of the tropical and subtropical Atlantic and Pacific oceans and highest in Circumpolar Deep Water (CDW) at 22°N in the far North Pacific, where deep waters have been out of contact with the atmosphere and thus sunlight for centuries (Figure 3A). Notably in the subtropical North Atlantic and the subtropical South Pacific, deplete *I*_photo_ values reached 1500 m (Figure 3A). The *I*_photo_ values in the whole data set had low correlations with both the *I*_bio_ index and the degradation index (Figure 4C,D; *R*^2^ = 0.3 for both regressions).

The *I*_bio_ values decreased with increasing density (Figure 2B). The *I*_bio_ value of the NEqPIW reference sample (0.19) is within the range of the *I*_bio_ values in the deep Atlantic and Pacific oceans (both had similar ranges of 0.15 < *I*_bio_ < 0.21; mean 0.18 ± 0.01). *I*_bio_ values in the whole data set significantly correlated with the molecular lability boundary (*R*^2^ = 0.81, Figure 4A)^26^ and *I*_deg_ (*R*^2^ = 0.89, Figure 4B).^28^ The range of *I*_bio_ values in the Southern Ocean was 0.15 to 0.23 (0.19 ± 0.01), like the range of *I*_bio_ values in the deep Atlantic and Pacific. In the surface Atlantic and Pacific, *I*_bio_ ranged from 0.24 to 0.30 (0.26 ± 0.02) and 0.20 to 0.29 (0.25 ± 0.02), respectively. Values for *I*_bio_ were highest in the warm and productive surface layers in the Atlantic (*I*_bio_ = 0.30; Figure 3B) and lowest in Pacific Deep Water at 2000 m at the equator (*I*_bio_ = 0.15; Figure 3B). The highest *I*_bio_ values in the deep ocean were in the south equatorial Atlantic (Figure 3B). In this region, the *I*_bio_ values ranged from 0.23 to 0.30 (0.27 ± 0.02). It is noteworthy that the highest calculated *I*_bio_ for the Atlantic was still significantly lower than the *I*_bio_ calculated for the mesocosm DOM (*I*_bio_ = 1.05).

## Discussion

### Derivation of *I*_photo_ and *I*_bio_ as Process-Specific Indices

We derived *I*_bio_ and *I*_photo_ from five selected “marker peaks” (Table 1) isolated from the mesocosm^22^ and photodegradation^14^ experimental data, respectively. These peaks covered a maximum possible mass range and are applicable in a maximum variety of different environments. When more peaks are chosen, there is a higher probability of one not being present in a sample of interest. Likewise, the *I*_deg_(28) is also based on 5 peaks. The wider mass range for *I*_photo_ than *I*_bio_ likely reflects that photolabile DOM compounds generally have a higher molecular weight than photoresistant compounds. In contrast, the most prominent signature of biological production and transformation of DOM is found in the lower mass range, especially when compared to refractory DOM.^22^

We considered creating indices for biological degradation and photoproduction that would complement the two indices in this work. However, due to experimental constraints in the mesocosm experiment and photoproduced molecular formulas not fitting the criteria of a molecular index, it was not possible to define respective process indices. In the mesocosm experiment, assessing degradation is impossible given the criteria of the indices, in which the molecular formulas would have to persist and be 5% of the maximum peak intensity of the reference sample. Biodegraded molecular formulas would be absent from most environmental samples. However, the *I*_deg_ gives a good indication of the degradation state of a DOM sample, because it identifies molecular formulas that have been already transformed from more labile to more recalcitrant DOM and correlate positively with increasing ^14^C age.^28^ Instead of being completely degraded, however, the molecular formulas in *I*_deg_ are the accumulation of more recalcitrant molecular constituents that are produced after a cascade of molecular formations and transformations over time and space. The *I*_bio_ index covers the formation and transformation of DOM from when it is photosynthetically produced to microbially modified over several years; this index is mostly an indicator of semilabile to semirefractory DOM, as it was derived from DOM samples from the second year of the mesocosm experiment.^22^ Its negative correlation with *I*_deg_ suggests that the chemical transformations in DOM as it transforms from more labile/semilabile DOM to more refractory results in a loss of the molecular formulas in *I*_bio_ and an accumulation of those in *I*_deg_.

Photoproduction of compounds in DOM does occur; 106 molecular formulas were photosynthetically produced after 28 days of irradiation in the original experiment.^14^ Of these 106 molecular formulas, 40 were present in our deep seawater reference, yet none of them fulfilled the first criteria, as they were all lower than 5% of the relative peak intensity of the maximum peak intensity of the standard. Therefore, while it does appear that a fraction of these photoproduced molecular formulas are present in the deep ocean, their abundances are low and not enough to be reproducibly detected and quantified as a molecular index.

Assuming our choice of endmembers (i.e., mesocosm DOM for bioformation and transformation and photodegraded NADW for photodegradation) covered a maximum range of possible changes in the molecular DOM composition, we calculated *I*_photo_ and the *I*_bio_ from their respective experimental data sets. Pure mesocosm DOM, which at the time of sampling consists mainly of semilabile and semirefractory DOM,^22^ has *I*_bio_ values >1, which are much higher than the *I*_bio_ in the most productive subtropical and tropical surface waters (0.3; Figure 3B). This difference implies that the freshly/recently produced DOM in the open ocean is diluted by more recalcitrant DOM ubiquitously present in marine DOM.^23^ Moreover, most freshly produced DOM in the open ocean has a very short turnover time;^1^ it is either taken up quickly by heterotrophic microorganisms or diluted by mixing water masses. The *I*_bio_ value of the mesocosm implies that in DOM production hot spots such as phytoplankton blooms or coastal areas, *I*_bio_ could be considerably higher than observed in the open ocean.

Remarkably, the *I*_photo_ values in the surface mixed layer of the subtropical Atlantic and Pacific oceans are even lower than the *I*_photo_ values of the experimentally photodegraded NADW DOM (Figure 2A; *I*_photo_ values below the blue box). The experiment in Stubbins and Dittmar^14^ consisted of 28 days of constant irradiation. In that study, 1 day of irradiation equals 1.27 times daily solar irradiance during winter in the subtropics or 0.67 times the daily (12 h) irradiance at the equator. Therefore, this study would equate to over two months of sun exposure in the winter subtropics and 38 days in the tropical surface ocean. In the summer subtropics and equator, however, DOM is exposed to high levels of irradiation for months to years, respectively. As all samples were collected in the summer, the lower *I*_photo_ values in the tropics and subtropics likely reflect their long exposure to sunlight. Moreover, Stubbins and Dittmar^14^ only looked at the photodegradation of deep water DOM. The photosusceptibility of freshly produced DOM in the subtropical surface ocean may likely be different than that of recalcitrant DOM in NADW. The composition of labile and semilabile DOM present in the subtropical surface ocean may render it more susceptible to photodegradation than the deep water DOM tested in Stubbins and Dittmar.^14^ Therefore, *I*_photo_ values of photodegraded, freshly produced DOM may intrinsically be lower than that of photodegraded, recalcitrant DOM. In a laboratory experiment coupling UV exposure of DOM with mesocosm incubations, the refractory constituents in the DOM were not substantially photochemically affected, suggesting that recalcitrant DOM is a result of both biological and abiotic reworking, and potentially less susceptible to photodegradation than semilabile DOM.^51^

### Using *I*_photo_ and *I*_bio_ to Distinguish Processes in the Marine Environment

**Figure 5:**
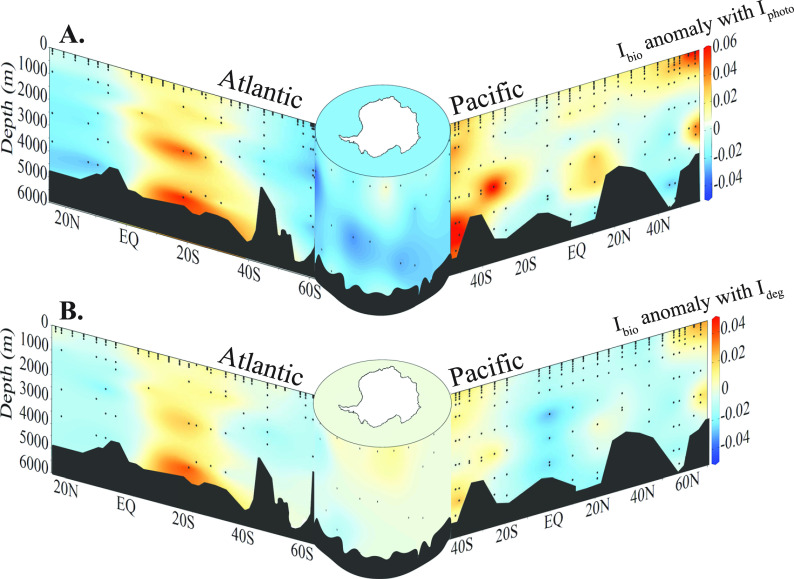
U-shaped plot of the anomaly of *I*_bio_ vs (A) *I*_photo_ and (B) *I*_deg_, based on the linear regression defined in Figure 4B and C. Here, an anomaly >0 indicates regions where measured *I*_bio_ is higher than would be expected if the two indices would covary. Sections are defined using cruises SO248, SO254, ANT 28-5, ANT28-4, and ANT28-2 (Figure 1).

By applying both the *I*_photo_ and the *I*_bio_ to a wide range of environmental samples, we demonstrate that these new indices are valuable tools for distinguishing two important processes that shape the molecular composition of oceanic DOM. Photodegradation and biological production and transformations have their maximum impact in the sunlit, warm, and productive surface layer (Figure 3). Therefore, the imprints of photodegradation and biological transformations on DOM composition often coexist but can also diverge. To assess in which regions of the global ocean *I*_bio_ and *I*_photo_ covary vs diverge, we calculated the anomaly of their regression (Figures 4C and 5). Where there is a high *I*_photo_, the linear model in Figure 4C predicts a low *I*_bio_; i.e., in most cases the two processes co-occur. However, there are regions with a divergence between the two indices. For example, the subtropical North Atlantic and the subtropical South Pacific exhibit low *I*_photo_ and relatively low *I*_bio_ values (Figures 3A and 5). The nutrient limitation in subtropical gyres could explain the low *I*_bio_ values there.

Moreover, subtropical gyres experience high levels of sunlight exposure which leaves a distinct photodegraded signature in DOM.^32^ Notably, however, the low *I*_photo_ values in these regions reach down to intermediate waters at 1500 m (Figure 3A), suggesting that the gyre is introducing photodegraded DOM to depth. Warm-core eddies associated with subtropical gyres can reach down to 1500 m and introduce oxygen and heat to these depths, and also photodegraded DOM.^52^ This signature with low *I*_photo_ is lost in waters outside the gyre, however, suggesting that the molecular signal of photodegraded DOM introduced to these depths is transient, i.e., removed, either by chemical processes or mixing with surrounding intermediate and deep waters.

Higher *I*_bio_ values are mostly restricted to the upper 200 m (Figure 3B). Because both semilabile and semirefractory DOM are persistent on time scales greater than one year, accumulation of these DOM fractions in the upper mixed layer is possible (Hansell, 2013) and their contribution to the overall DOM pool is detectable with *I*_bio_. However, as these DOM fractions sustain the subsurface microbial loop in the mesopelagic, they are mostly remineralized before reaching the deep ocean.^53,54^ Consequently, *I*_bio_ decreases from ∼0.3 to ≤0.2 below the surface mixed layer and remains low mostly throughout the deep ocean (Figure 3B). Nevertheless, there are localized events that can export particle-derived semilabile DOM into the bathypelagic.^55^ At ∼10° S in the Atlantic, we observed elevated *I*_bio_ values at depths near the Brazil-Malvinas Confluence zone.

Notably at depth where *I*_bio_ is elevated, *I*_photo_ follows a similar trend (Figure 3). For instance, near the shelf edge in the Pacific sector of the Southern Ocean, elevated *I*_bio_ values indicate a higher contribution of microbially produced DOM originating from the Antarctic shelf advected into deep waters. These samples also hold elevated *I*_photo_ values when compared to those in deep waters in the open Atlantic and Pacific oceans. In the waters surrounding Antarctica, low, seasonal solar irradiance would prevent the DOM there from extensive photodegradation. Moreover, the Antarctic circumpolar current and rapid advection of surface waters to depth would move the surface waters there to depth more quickly, carrying with it a moderate *I*_photo_ signature (Figure 3A).

The South equatorial Atlantic also holds higher *I*_photo_ values at >1000 m depth. In the deep North Pacific near 20° and 50°N, there are two regions with elevated *I*_bio_ and *I*_photo_ values (Figure 3A and B). The higher *I*_bio_ co-occurring with higher *I*_photo_ at depth implies that in these regions, DOM produced at the surface was removed prior to photodegradation due to either rapid advection or particle export. Finally, the *I*_photo_ values in the deep Pacific are generally higher than those in the deep Atlantic (Figure 3A). The generally higher *I*_photo_ values in the deep Pacific are consistent with the accumulation of CDOM there. The deep Atlantic has younger water masses that would hold lower *I*_photo_ values, as the deep waters there have been in more recent contact with the sunlit ocean.

### Relating *I*_bio_ and *I*_photo_ to *I*_deg_

Previously published indices based on FT-ICR-MS molecular data describing the state of a given DOM sample were derived by correlating intensities of mass peaks with specific sample characteristics like radiocarbon age^28^ or fraction of terrestrial material^33^ (δ^13^C). These previously published indices are based on “marker peaks” that change systematically with the chosen parameters. Both *I*_bio_ and *I*_photo_ are also based on distinct “marker peaks”, but rather than describing the current state of the DOM composition, these novel indices reveal the dominant processes that led to this current state.

The degradation index (*I*_deg_; Figure 3C) assesses the degradation state of a DOM sample based on the relative peak intensities of molecular formulas that correlated with apparent ^14^C age in Flerus et al.^28^ The more degraded (i.e., older) a sample is, the higher the resulting *I*_deg_. There was a highly negative correlation between *I*_deg_ and *I*_bio_ in our data set (*R*^2^ = 0.89; Figure 4B). When a detectable biological signature is present, *I*_deg_ and *I*_bio_ provide complementary information about the degradation state of DOM and the contribution of DOM produced by microbial communities. For instance, in the mesocosm, *I*_deg_ is 0.15, indicating that the DOM is minimally degraded. In that same sample, the *I*_bio_ is 1.05, suggesting that essentially all of the material is biologically produced. In deep waters (and our NEqPIW reference), *I*_deg_ is ∼0.8 (Figure 3C) and *I*_bio_ is ∼0.2 (Figure 3B).

However, *I*_bio_ provides information on microbially produced DOM that is not revealed by *I*_deg_. The anomaly of the regression of *I*_deg_ vs *I*_bio_ (Figures 4B and 5B) illustrates the specific regions where microbial production of DOM is not reflected in its bulk degradation state. Antarctic shelf systems have large seasonal phytoplankton blooms coupled with extensive CO_2_ drawdowns.^56^ However, the DOM produced on Antarctic shelves is largely respired upon export into the deep Southern Ocean.^57^ The *I*_deg_ distribution in the Southern Ocean likewise reflects those findings, as the DOM has similar *I*_deg_ values in the Southern Ocean and bottom waters in the Atlantic and Pacific oceans. However, the positive anomaly of *I*_bio_ there suggests that even though the DOM there appears degraded, there is a more recent microbial signature, implying microbial reworking and renewing of DOM that is exported into Southern Ocean derived deep and bottom waters. Recent work using the same data set^49^ found that the Southern Ocean and North Pacific (areas of deep water mass upwelling) are a sink of refractory DOM.^58^ The elevated anomaly in *I*_bio_ in the Pacific Sector of the Southern Ocean and in the far North Pacific supports this finding. Even though there were no substantial changes in carbon concentration there, the DOM from the deep ocean was evidently microbially reworked into a more semilabile form once it reached the surface ocean.

The weak correlations between *I*_photo_ and *I*_deg_ and *I*_photo_ and *I*_bio_ (Figure 4C and D) indicate that photodegradation, age-related degradation, and bioformation and transformation are geographically unrelated processes. Moreover, the *I*_deg_ of the photodegradation data set^14^ remained stable during photodegradation (0.86 and 0.82 before and after irradiation, respectively), illustrating that photodegradation is not the major process driving changes in *I*_deg_. Based on this finding, we conclude that *I*_photo_ is not biased by other degradation processes, but instead is a unique indicator for both regions of photodegradation (i.e., subtropical gyres; Figure 3A) and accumulation of photolabile DOM (i.e., far North Pacific, deep Atlantic; Figure 3A). In general, DOM that undergoes extensive photodegradation, as observed in the sun-lit surface ocean, holds low *I*_photo_ values. These low values are present down to 1500 m water depth in the subtropics, suggesting that photodegraded DOM is injected into the mesopelagic in subtropical gyres. Future work applying these indices to coasts, lakes, streams, rivers, and sediment porewaters will provide further insights as to whether these indices can be applied to samples beyond the marine environment and the extent to which these processes drive DOM molecular composition in each of these environments.

### Summary

This study introduces two indices (*I*_bio_ and *I*_photo_) to distinguish the extent to which microbial reworking and photodegradation shape the DOM composition in the global ocean. The process indicators are derived from controlled laboratory experiments, as the distinction between processes and the development of process-related indices is not achievable in the natural environment. Both *I*_bio_ and *I*_photo_ are novel tools for assessing the specific processes behind observed changes in the natural DOM composition. When applied to a global ocean data set, these indices disclose the extent to which each respective process plays a role in the marine environment, and where they covary vs diverge. For instance, DOM composition data at depth with higher *I*_bio_ values also has higher *I*_photo_ values, suggesting that microbially produced DOM includes components that are susceptible to photodegradation at the surface ocean but remain in the deep ocean. Moreover, subtropical gyres appear to inject photodegraded DOM into depths up to 1500 m; this feature is absent in the *I*_bio_ signature, as most labile forms of DOM are removed by ∼200 m depth.^2^ The higher *I*_bio_ anomalies with *I*_deg_ in the Southern Ocean and far North Pacific imply that the DOM there has a relatively recent microbial signature, likely due to reworking of recalcitrant DOM by microbial communities in these regions of deep water overturning. The information provided by both indices is crucial for disentangling the mechanisms controlling the molecular composition of DOM and is therefore relevant to fully understand the turnover of this large carbon reservoir on a global scale.
